# Health research capacity of professional and technical personnel in a first-class tertiary hospital in northwest China: multilevel repeated measurement, 2013–2017, a pilot study

**DOI:** 10.1186/s12961-020-00616-7

**Published:** 2020-09-17

**Authors:** Peijing Yan, Yongfeng Lao, Zhenxing Lu, Xu Hui, Biao Zhou, Xinyu Zhu, Xiaojie Chen, Li Li, Zixuan Wang, Min Zhang, Kehu Yang

**Affiliations:** 1 Institute of Clinical Research and Evidence-Based Medicine, Gansu Provincial Hospital, Lanzhou, 730000 China; 2 Department of Epidemiology and Health Statistics, West China School of Public Health and West China Fourth Hospital, Sichuan University, Chengdu, 610044 China; 3 Second Clinical Medical College, Lanzhou University, Lanzhou, 730000 China; 4 Institute of Medical Research, Northwestern Polytechnical University, Northwestern Polytechnical University, 710000 China; 5 School of Public Health, Lanzhou University, Lanzhou, 730000 China; 6 Department of Scientific Research, Gansu Provincial Hospital, Lanzhou, 730000 China; 7 Evidence-Based Medicine Center, School of Basic Medical Sciences, Lanzhou University, Lanzhou, 730000 China; 8 Evidence Based Social Science Research Center, School of Public Health, Lanzhou University, Lanzhou, 730000 China; 9 Key Laboratory of Evidence Based Medicine and Knowledge Translation of Gansu Province, Lanzhou, 730000 China

**Keywords:** Health research capacity; professional and technical personnel; Chinese hospital; multilevel repeated measurement

## Abstract

**Objectives:**

To explore the health research capacity (HRC) and factors associated with professional and technical personnel (PTP) in a first-class tertiary hospital in northwest China.

**Methods:**

We collected the repeated measurement data from a first-class tertiary hospital in northwest China between 2013 and 2017. HRC of PTP was assessed by a comprehensive evaluation system and measured by research capacity score (RCS). The participants were divided into research group (RCS >0) and comparison group (RCS = 0); participants of the comparison group were selected by two-stage stratified random sampling. Multilevel model for repeated measures was used to investigate the potential factors associated with HRC.

**Results:**

A total of 924 PTP were included (308 in the research group and 616 in the comparison group). This study found consistent growth in RCS and associated 95% CIs for the hospital during 2013 and 2017. The linear multilevel model showed PTP with a doctorate degree had higher RCS than those with a master’s degree (β, 1.74; *P* <0.001), bachelor’s degree (β, 2.02; *P *<0.001) and others without a degree (β, 2.32; *P* <0.001). Furthermore, the PTP with intermediate (β, 0.13; *P *= 0.015), vice-high (β, 0.27; *P* = 0.001) and senior (β, 0.63; *P* <0.001) professional titles had higher RCS than those with junior positions. Compared with PTP in the administration, those in paediatrics had higher RCS (β, 0.28; *P* = 0.047) though similar to PTP in other departments. PTP with an administrative position had a higher RCS than those in non-administrative positions (β, 0.26; *P* <0.001). The RCS increased with the research fund (β, 0.15; *P* <0.001). However, no associations were found between RCS and sex, age, ethnic, graduate school or technical type.

**Conclusions:**

HRC with associated variation of PTP for the hospital in northwest China increasingly improved and degree, professional title, administrative position, and research fund were related to HRC of PTP. Multi-central prospective studies are needed to clarify the potential relationship of related factors and HRC of PTP.

## Introduction

Health research provides new knowledge that can be transferred into practice [1], creates advanced care environments [2], enhances the support available to patients and their families [3], and has been described as the missing link in the development of high-quality, evidence-based healthcare for the population [4]. Research outputs from hospitals have grown faster than those from universities, especially since 2006/2007 [5] and hospitals engaged in research have been recognised as providing better patient care [6]. Physician-scientists play a unique and critical role in health research [7]. Furthermore, with evidence-based practice spreading worldwide, professional and technical personnel (PTP) are responsible for delivering high-quality care based on the best evidence [8]. Therefore, the health research capacity (HRC) of PTP plays vital role in health research.

In recent years, studies on HRC have focused on nursing [9–11], midwifery [12], pharmacists [13] and other healthcare areas. However, developing high-quality hospital-based research is needed in our health system as well as for various PTP [14]. In addition, different approaches and indexes have been used to assess the HRC for nursing [10, 15–17] and salary sources, research funding, time spent in research, bibliometric analyses and other non-comprehensive indexes have been used to evaluate the HRC among other PTPs [10, 18–20]. However, there is no standardised method for measuring research capacity [18].

Based on a hospital's ability to provide medical care, medical education and conduct health research, hospitals in China are designated as primary, secondary or tertiary institutions [21]. Tertiary hospitals perform a larger role with regard to medical education and scientific research and serve as medical hubs providing care to multiple regions and are superior to primary hospitals and secondary hospitals [22, 23]. Furthermore, based on the level of service provision, size, medical technology, medical equipment, and management and medical quality, these three grades are further subdivided into three subsidiary levels, resulting in a total of nine levels. A first-class tertiary hospital is more specialized than any other level.

To date, no research has focused on hospitals of northwest China, where the HRC of PTP is in its initial and developmental stage. Therefore, herein, we chose all of the PTP in a Chinese first-class tertiary hospital of northwest China as the research objective. Health delivery systems vary considerably between different countries; thus, the HRC was assessed by a comprehensive evaluation system specific for China [24]. Additionally, some studies in HRC among other medical staff have been cross-sectional studies [2, 25], whereas others have used multiple linear regression analysis [26, 27], which ignores delayed effects [28] and long-term nature of scientific research [29]. Therefore, this study adopted the multilevel repeated measurement model [30, 31] to explore the HRC and factors associated with all PTP in a first-class tertiary hospital in northwest China.

## Methods

### Data source

The present study was based on data of the official figures from a first-class tertiary hospital in northwest China. Ethics approval was granted by the Institutional Ethics Committee of the Gansu Provincial Hospital. We selected PTP of the hospital as the research objective and collected the characteristics of participants and the information of HRC between 2013 and 2017.

### Assessment of HRC

HRC was assessed by a comprehensive evaluation system and measured by research capacity score (RCS). The evaluation system was adopted by Hongwei Fan et al. in 2015 [24, 32, 33] based on the Delphi method and the selected 38 experts have rich experience in scientific research management, basic research, clinical research and other fields. The evaluation system covers the information of research capacity with associated weight score. After two rounds of the Delphi consulting, the scientific evaluation system was established and included 6 first-level indicators and 28 second-level indicators. The details of the evaluation system are included in Additional file 1. The mean of expert authority coefficients was 0.879, the Kendall's W coefficient of two rounds of the Delphi consulting were 0.79 and 0.83 [24]. The evaluation system had good reliability and validity (Cronbach’s α = 0.950) [32]. The information of research capacity contains obtained research projects, research awards, patents, published scientific papers, monographs and the number of trained students [24].

The RCS of participants is equal to the summary of the weight score in HRC. Considering the delayed effect [28] and long-term nature of scientific research [29], this study collected the RCS of the participants per year from 2013 to 2017.

### Participants and randomisation

This study divided the participants into the research group (*n* = 308) and a comparison group (*n* = 1871) based on the RCS. The PTP whose RCS was greater than zero in the 5-year period belonged to the research group and the other participants belonged to the comparison group. The proportion of degree types (doctor, nurse, technician, pharmacists and other PTP) of all the PTP for the hospital was different from those of the research group and there were significant differences among technical types in sex, degree and other factors. To balance the number of participants between the two groups and reduce the potential bias caused by the different characteristics of PTP, a two-stage stratified random sampling method was adopted to determine the comparison group.

**Fig. 1:**
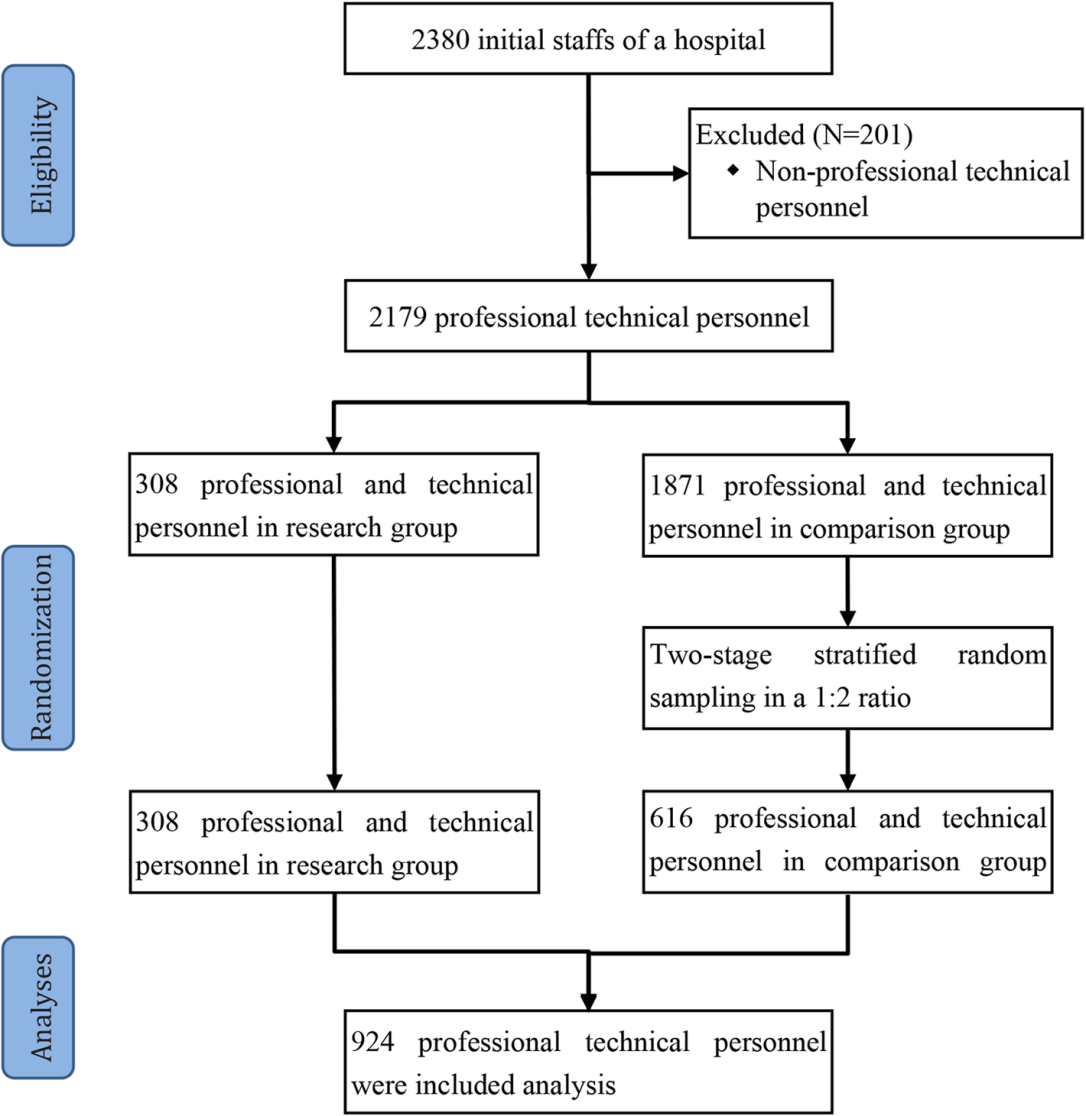
Sampling and ascertainment of professional and technical personnel

Two-stage stratified random sampling was performed as follows: (1) the PTP in the comparison group were stratified into five levels based on the degree type of research group: doctor, nurse, technician, pharmacists and other PTP; and (2) we selected each level in a 1:2 ratio (research group versus comparison group), and 616 participants were randomly selected from the comparison group as the research objective. Therefore, a total sample of 924 professional technical personnel was included in this study. Figure 1 shows the flow of PTP sampling and ascertainment.

### Data management

We developed a study manual of operations to standardise all screening and data management, which was used to train all researchers in this study.

The information of participant-level characteristics included basic demographic characteristics (sex, age, ethnic, birthplace, etc.); the latest degree information (highest degree, graduate school, length since graduation, etc.); and the latest work information (department, technical type, title, whether in an administrative position or not, etc.). The repeated measurement of scientific research information (in the 5 years 2013–2017) included the number of obtained research projects, research awards, research fund or patents; published scientific papers or monographs, and the number of trained students.

Highest degree containing doctor, master, bachelor and others (without a degree). Graduate school of PTP includes foreign university, 985/211 university, common university, and others. (985/211 university means one of the universities participating in either Project 211 or Project 985, financed by the government of the People’s Republic of China to build world-class universities for the 21st century; universities in projects 985 or 211 are first class universities in China [34].) Common university means the full-time university besides a foreign university or a 985/211 university, and other universities containing adult education, network education and other various part-time courses. Degree type includes doctors, nurses, medical technicians, pharmacists and others (other professional and technical types such as accountant, economist, etc.).

### Statistical analysis

For continuous variables, data were reported either as the mean ± standard deviation (SD) or the median with interquartile range (IQR) according to normality tests (Shapiro–Wilk and Shapiro–Francia tests for normality). Categorical variables were expressed as frequencies and percentages. Pearson's χ^2^, two-sample *t* test and Kruskal–Wallis rank test were used to compare the difference of participant-level characteristics between the two groups.

Taking delayed effect [28] and long-term of scientific research [28] into account, we repeated the measurement of research capacity of PTP from 2013 to 2017. Thus, the traditional multiple regression model is not suitable. Furthermore, repeated measurement ANOVA could not handle the covariates well as it regarded random effects as useless parameters, which leads to information loss [35]. Therefore, we used multilevel models for repeated measures to investigate the potential factors associated with HRC for PTP and to accommodate data structured hierarchically [31]. We used linear multilevel models with random intercepts for continuous measures of HRC, and a two-level variance structure with repeated assessment measures nested within PTP. Multicollinearity in the predictors can affect the results of multilevel models. Multilevel variance inflation factor, a form of analysis used in multilevel modelling, was used to assess multicollinearity among predictors; variables with multilevel variance inflation factor values over 10 indicate potential collinearity problems [36–38]. All of the main effects and interactions were assessed using *P* values from Wald tests.

Covariates included in the final model were (1) sex, age and ethnicity for basic demographic characteristics; (2) highest degree and graduate school for degree information; (3) technical type, title, department, whether holding an administrative position or not for work information; and (4) the amount of research funded each year between 2013 and 2017 for research information.

Data from these analyses were reported as coefficients with 95% confidence intervals (CIs). For all statistical tests, a two-tailed a level of 0.05 was used. Statistical analyses and preparation of figures were performed using Stata, version 13.0 (Stata Corp., College Station, TX, USA) and MLwiN, version 2.30.

## Results

### Characteristics of participants

A total of 924 PTP were included (308 in the research group, 616 in the comparison group). The average age was 44.84 ± 8.51 years for the PTP in the research group and 37.71 ± 8.90 years for the comparison group. Approximately 44.26% (409) of participants were male.

Table 1 shows the participant-level characteristics in the research and comparison groups. The HRC were significantly different among participants of different ages (*P* <0.001), highest degree (*P* <0.001), graduate school (*P* = 0.003), length since graduation (*P* <0.001), professional title (*P* <0.001), administrative position (*P* <0.001) and research funding (*P* <0.001) between the two groups. However, no differences were found in sex, ethnicity and other participant-level characteristics.

**Table 1:** Participant-level characteristics in the two groups

Characteristics	Comparison group		Research group		*P* value
	*n*	(%)	*N*	(%)	
Sex					0.174
Male	263	42.69	146	47.40	
Female	353	57.31	162	52.60	
Ethnic					0.279
Minority	27	4.38	9	2.92	
Han	589	95.62	299	97.08	
Degree					<0.001
Doctor	3	0.49	64	20.78	
Master	404	65.58	142	46.10	
Bachelor	102	16.56	59	19.16	
Others	107	17.37	43	13.96	
Graduate school					0.003
985/211 University	281	45.62	159	51.62	
Foreign University	0	0.00	5	1.62	
Common University	226	36.69	98	31.82	
Others	109	17.69	46	14.94	
Degree type					1.000
Doctor	452	73.38	226	73.38	
Nurse	64	10.39	32	10.39	
Medical	54	8.77	27	8.77	
Pharmacist	34	5.52	17	5.52	
Others	12	1.95	6	1.95	
Title					<0.001
Junior	302	49.03	23	7.47	
Intermediate	200	32.47	85	27.60	
Vice-High	80	12.99	100	32.47	
Senior	34	5.52	100	32.47	
Department					0.589
Administration	50	8.12	31	10.06	
Medicine	151	24.51	72	23.38	
Surgery	176	28.57	91	29.55	
Obstetric-Gynaecology	27	4.38	9	2.92	
Paediatrics	18	2.92	4	1.30	
Medical technician	187	30.36	98	31.82	
Ophthalmology	7	1.14	3	0.97	
In administrative position					<0.001
No	573	93.02	170	55.19	
Yes	43	6.98	138	44.81	
Age (Year), Mean ± SD	37.71 ± 8.90		44.84 ± 8.51		<0.001
Length since graduation (year)^a^	7.14 (7.58)		11.14 (7.09)		<0.001
Research fund (CNY, 10,000)^a^	0.00 (0.00)		0.00 (0.00)		<0.001

^a^Measured by medium (Inter Quartile Range) and tested by Kruskal–Wallis rank test

### The trend of HRC over time

**Fig. 2:**
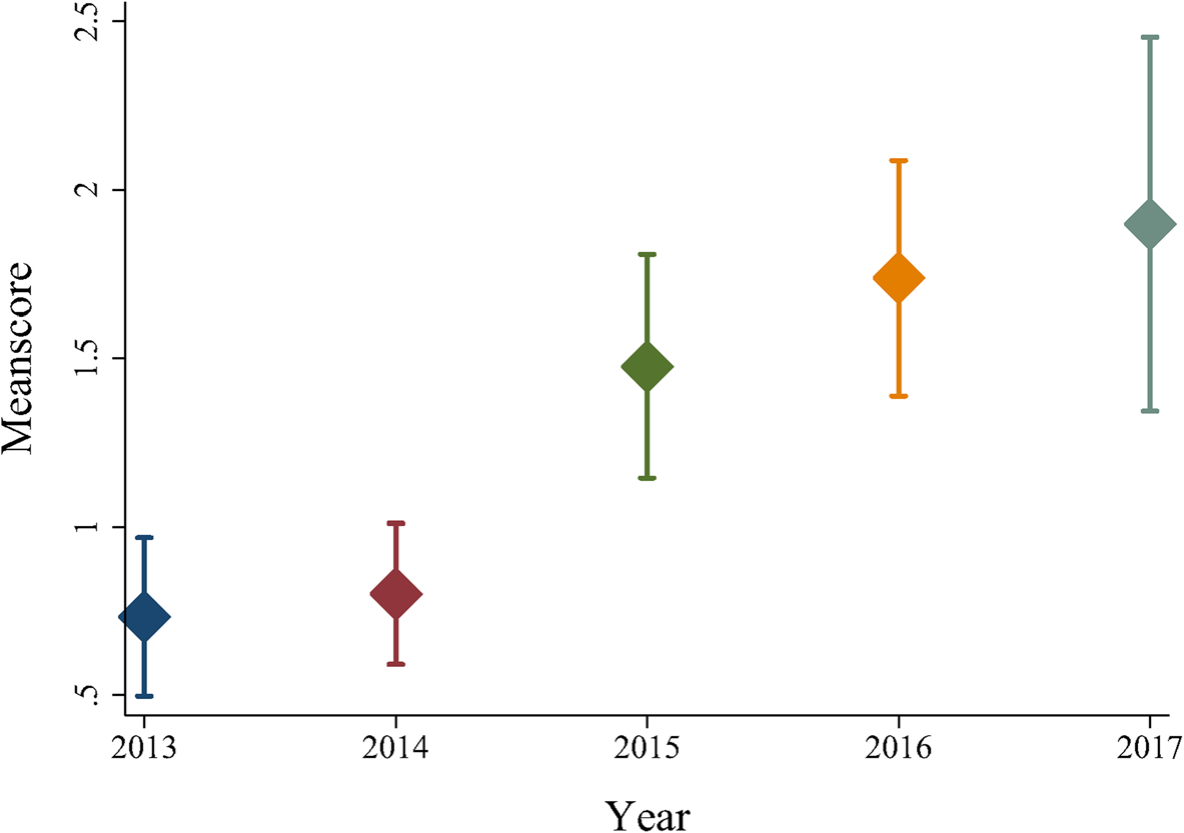
The trend of health research capacity for all participants

Figure 2 shows the trend of health RCS with the associated 95% CIs of the first-class tertiary hospital from 2013 to 2017. The results showed consistent growth in RCS during 2013 and 2017, and there was a sharp rise in 2015. In addition, although the number of PTP in the hospital had increased gradually over the past 5 years, the 95% CIs of the RCS widened significantly, indicating that the variation in the HRC of the PTP increased dramatically.

### HRC at participant level

**Fig. 3:**
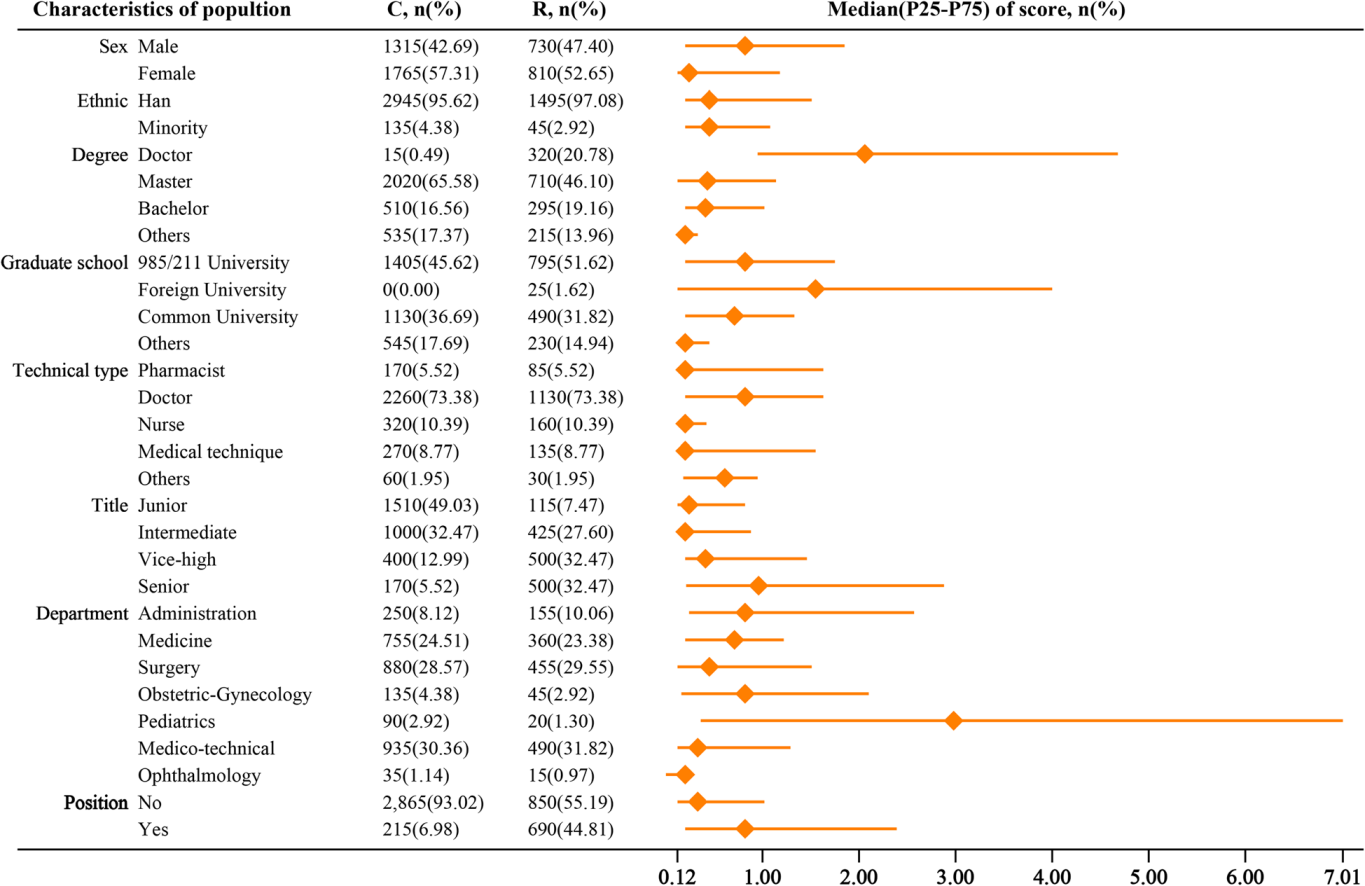
The research capacity score of participants stratified by characteristics*. *: C, Comparison group; R, Research group; P_25_, the first quartile; P_75_, the third quartile; Position, in administrative positions

Figure 3 shows the median P_25_ and P_75_ of RCS of PTP with different characteristics. The results showed that men PTP had higher RCS but greater IQR than women PTP; the Han nationality and minority participants had similar RCS, but the RCS of Han nationality participants had bigger IQR; RCS increased with the educational degree of participants, but IQR decreased with the educational degree of participants; those graduated from foreign universities had the highest RCS but greatest IQR than others, followed by those graduated from 985/211 universities, common universities and other schools.

The participants with doctorate degrees had the highest RCS and those with pharmacist, nurse and medical degrees had similar RCS, but participants with doctor and pharmacist degrees had the greatest IQR and those with nursing degrees had the smallest IQR. Participants with senior titles had the highest RCS, followed by those with vice-high, junior and intermediate title, though participants with senior titles had the greatest IQR, followed by those with vice-high, intermediate and junior title. Participants in paediatrics had the highest RCS but the greatest IQR than others. Finally, those in administrative positions had higher RCS but greater IQR than those in non-administrative positions.

### Multilevel repeated measurement model for HRC

**Fig. 4:**
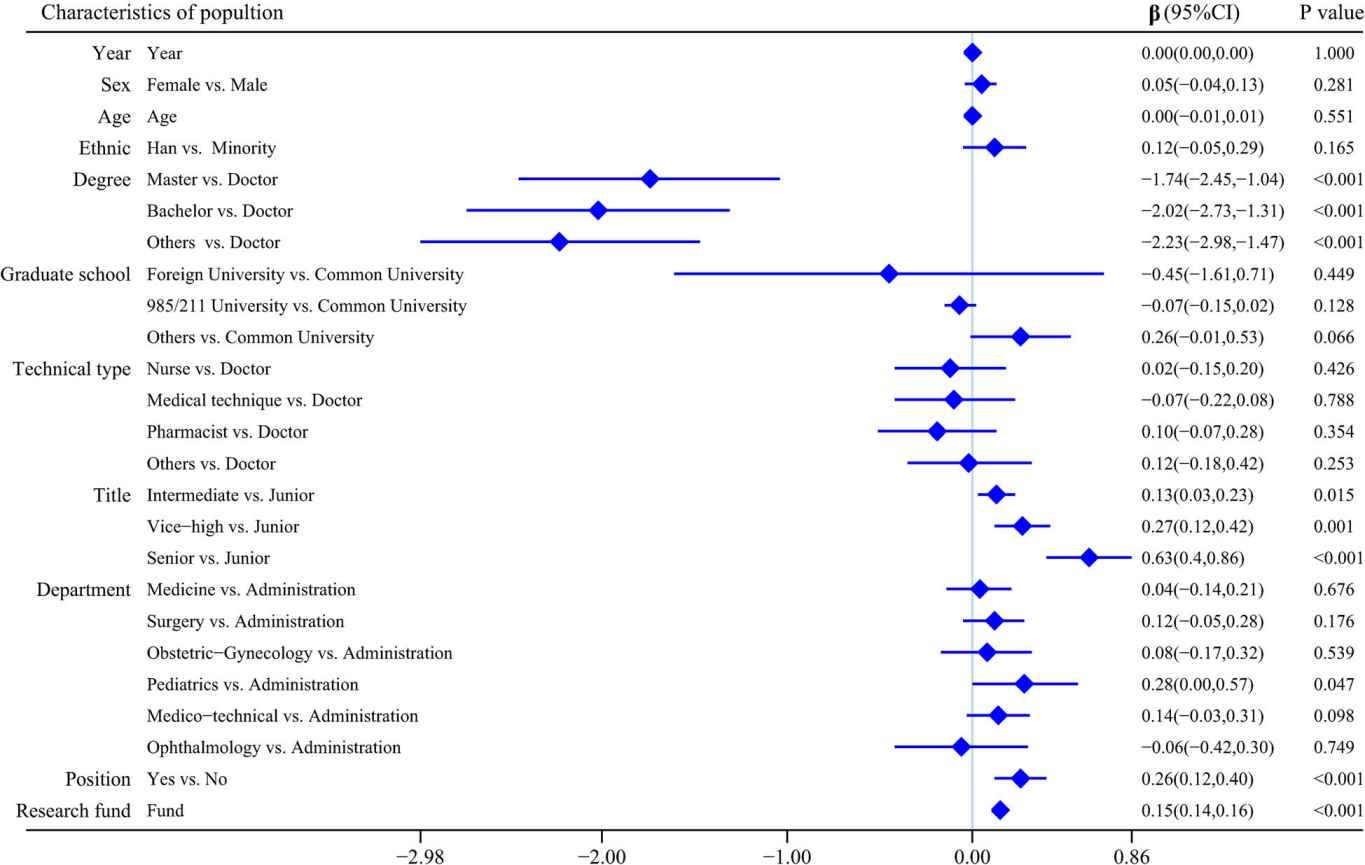
Two-level repeated measurement model for the factors associated with health research capacity

Figure 4 presents the results of the multilevel repeated measurement model for the factors associated with HRC of PTP. All of the multilevel variance inflation factors ranged from 1.018 to 4.357 (details are included in Additional file 2), suggesting the absence of potential collinearity problems with these results. After controlling for the potential confounders, the HRC of PTP was related to degree, professional title, department, whether in an administrative position and research fund.

Participants with a doctorate’s degree had higher RCS than those with a master’s degree (β, 1.74; 95% CI 1.04–2.45; *P* <0.001), bachelor’s degree (β, 2.02; 95% CI 1.31–2.73; *P* <0.001) and others without a degree (β, 2.32; 95% CI 1.47–2.98; *P* <0.001). Furthermore, participants with intermediate (*P* = 0.015), vice-high (*P* = 0.001) and senior (*P* <0.001) professional titles had 0.13 (95% CI 0.03–0.23) points, 0.27 (95% CI 0.12–0.42) points and 0.63 (95% CI 0.40–0.80) points more than those with junior title on average. Compared with participants in administrative roles, those in paediatrics had higher RCS (β, 0.28; 95% CI 0.00–0.57; *P* = 0.047) but similar to those in other departments. Participants in administrative positions had a higher RCS than those in non-administrative positions (β, 0.26; 95% CI 0.12–0.40; *P* <0.001). The RCS increased with the research fund (β, 0.15; 95% CI 0.14–0.16; *P* <0.001). However, no associations were found between RCS and sex, age, ethnicity, graduate school or technical type of participants.

## Discussion

This study found steady growth in HRC of the first-class tertiary hospital during 2013 and 2017, with a sharp rise in 2015. In addition, the HRC of PTP increased with degree, professional title and research fund; those in administrative positions had better HRC than those in non-administrative positions. However, no association was found between graduate school and HRC.

The first-class tertiary hospital showed consistent growth in the HRC with associated variation during 2013 and 2017. Some factors might explain the consistent growth of HRC. Firstly, hospital managers have realised the importance of scientific research; thus, some internal incentives were used to promote research [39, 40]. Secondly, the PTP have undergone scientific training to improve their HRC [41, 42]. Thirdly, talents with excellent HRC have been recruited in hospital; however, this may also increase the variation in the HRC of PTP as recruited talents with superior capacity and positive attitude towards research would more easily gain more academic resources and subsequently improve the HRC. Such a trend seemed similar to Matthew effect [43], leading to increasing internal differences.

This study found that the HRC of PTP increased with the academic degree of PTP. Previous research in young Japanese nursing faculty showed that a doctorate degree was the most vital predictor of the number of publications [44] and researchers found that master’s degree was the crucial factor for research participation for nurses [45], similar to what we have observed herein because more academic training and research sources are critical components of the development of a research career [46]. Since participants who graduate from better universities will have better education and research resources and may carry this on to their working environments [47]; however, we found no correlation between graduate school and HRC. A study found that the training and work environments but not the prestige of their doctoral institution drove researcher productivity [48], similar to that observed herein. Degree and graduate school were often used as a measure of scientific capacity [49]; therefore, our results may provide a perspective for hospital administrators for what may be considered when bringing in talents and improving hospital research capacity.

A positive correlation was found between research funding and HRC of PTP, similar to a previous study suggesting a positive correlation between the funding support and research success [20]. As reported in previous studies, limited funding was one of the highest-ranking barriers to conducting research [50] and restricted collaboration between institutions [51]. Furthermore, the better funding support is a potential factor to build capacity of research [52, 53]. In order to obtain long-term and adequate research funding, researchers may be encouraged to set research agenda, focused on important issues and collaboration [54, 55], and then strengthed the HRC. A study in Bangladesh showed that the provision of un-earmarked core funding may be a potential mechanism for building research capacity [56]. On the other hand, PTP with higher HRC are more likely to get research funding, for example, attracting supported fellows to obtain additional research funding [20].

The HRC increased with the professional title of participants. Similar to our results, a previous study found a positive correlation between professional titles and publications in traditional Chinese medicine clinical investigators [50]. Participants with superior research capacity more easily met the requirements of professional title promotion and higher professional titles may bring more academic resources, including funding [57, 58]. Furthermore, research funding is related to higher HRC as this study and previous studies have found [54, 55]. Therefore, there may be a mutual incentive effect between professional titles and HRC, and the interaction is worth further exploration.

A previous study found that holding an administrative position had no effect on the academic productivity in a university hospital radiology department [59]; this is in contrast to our study, which suggested that personnel in administrative positions showed better research capacity than those in non-administrative positions in a Chinese hospital. Firstly, the research hospital in this study is located in the northwest of China, and therefore the ‘steel bowl’ model still has an influence on the hospital. The ‘steel bowl’ model in hospitals can be summarised as follows [60]: (1) PTP are hired in tenure and enjoy lifetime job security and (2) salaries are set by the government regardless of the individual’s performance. Thus, most of the PTP lack motivation to persist; however, PTP in administrative positions are more competitive than those in non-administrative positions. Secondly, PTP in administrative positions hold stronger attitudes and knowledge/skills on evidence-based practice [61]. Thirdly, the support and dedication of administrative superiors are key factors of the excellent teamwork [62] and teamwork plays a vital role on research. Compared with PTP in non-administrative positions, those in administrative positions can obtain more support and dedication.

### Strengths and limitations

This policy study was the first study on HRC and associated factors of PTP in a first-class tertiary hospital in northwest China. Furthermore, we used repeated measurement to handle the delayed effect and long-term characteristics of scientific research. However, there are some limitations to this study. Firstly, the nature of the study design, a retrospective study, will result in some biases and inaccuracies. In addition, for a retrospective design, the information in degree and work of participants during 2013–2017 was uncomplete, thus we chose the latest information. However, we used two-stage stratified random sampling to reduce the effect of information loss. Secondly, the results of this single-centre from northwest China, where the HRC of PTP is in its initial and developmental stage, might not be applicable to other clinical centres where the HRC of PTP is mature. Thirdly, the training, time spent on scientific research and attitudes towards research might influence the research capacity yet we could not collect such information. Fourthly, although the evaluation system this study adopted had good reliability and validity, the system was based on linear weight method, which may be difficult to objectively evaluate the scientific research capacity [63]. Finally, the proportion of the comparison group (RCS = 0) may influence on the result, but the data of this study does not apply to logistic multilevel repeated measurement model, thus we chose the linear multilevel model only.

## Conclusion

HRC with associated variation of PTP for the hospital in northwest China increasingly improved in the past 5 years and the degree, professional title, administrative position and research fund were related to HRC of PTP. Multi-central prospective studies are needed to clarify the potential relationship of related factors and HRC of PTP.

## Supplementary information

**Additional file 1: eTable 1** Scientific research capability evaluation system.

**Additional file 2: eTable 2** Multilevel variance inflation factors of variables in the multilevel repeated measurement model.

## Data Availability

The datasets used and analysed during the current study are available from the corresponding author on reasonable request.

## Abbreviations

HRC: health research capacity
PTP: professional and technical personnel
RCS: research capacity score
IQR: interquartile range
CIs: confidence intervals

## References

[1] Grimshaw JM, Eccles M, Lavis JN, Hill SJ, Squires JE (2012). Knowledge translation of research findings. Implementation Sci.

[2] Caminiti C, Iezzi E, Ghetti C, De’ Angelis G, Ferrari C (2015). A method for measuring individual research productivity in hospitals: development and feasibility. BMC Health Serv Res.

[3] Research in the behavioural and social sciences to improve cancer control and care: a strategy for development. A report of an expert group. European J Cancer 2004, 40(3):316–25. 10.1016/s0959-8049(03)00666-x 14746848

[4] Zwar NA, Weller DP, McCloughan L, Traynor VJ (2006). Supporting research in primary care: are practice-based research networks the missing link?. Med J Aust.

[5] Tijssen RJ, Winnink J (2016). Twenty-first century macro-trends in the institutional fabric of science: bibliometric monitoring and analysis. Scientometrics.

[6] Whitworth A, Haining S, Stringer H (2012). Enhancing research capacity across healthcare and higher education sectors: development and evaluation of an integrated model. BMC Health Serv Res.

[7] Ley TJ, Rosenberg LE (2005). The physician-scientist career pipeline in 2005. JAMA.

[8] Chen Q, Sun M, Tang S, Castro AR (2019). Research capacity in nursing: a concept analysis based on a scoping review. BMJ Open.

[9] McKee G, Codd M, Dempsey O, Gallagher P, Comiskey C (2017). Describing the implementation of an innovative intervention and evaluating its effectiveness in increasing research capacity of advanced clinical nurses: using the consolidated framework for implementation research. BMC Nurs.

[10] Gullick JG, West SH (2016). Building research capacity and productivity among advanced practice nurses: an evaluation of the Community of Practice model. J Adv Nurs.

[11] Squires A. US nursing and midwifery research capacity building opportunities to achieve the United Nations sustainable development goals. Nursing Outlook. 2019;67(6):642–48. 10.1016/j.outlook.2019.06.016 31376985

[12] Hauck YL, Lewis L, Bayes S, Keyes L (2015). Research capacity building in midwifery: Case study of an Australian Graduate Midwifery Research Intern Programme. Women Birth.

[13] Nazer LH, Tuffaha H, Jaddoua S (2017). A program to increase research productivity among hospital pharmacists. J Pharm Pract.

[14] Hong J, Xu J, Sun X (2013). Young doctors and the pressure of publication. Lancet.

[15] Bäck-Pettersson S, Jensen KP, Kylén S, Sernert N, Hermansson E (2013). Nurses’ experiences of participation in a research and development programme. J Clin Nurs.

[16] Corchon S, Portillo MC, Watson R, Saracibar M (2011). Nursing research capacity building in a Spanish hospital: an intervention study. J Clin Nurs.

[17] Duffy JR, Culp S, Sand-Jecklin K, Stroupe L, Lucke-Wold N (2016). Nurses’ research capacity, use of evidence, and research productivity in acute care: year 1 findings from a partnership study. J Nurs Admin.

[18] Embi PJ, Tsevat J (2012). Commentary: the relative research unit: providing incentives for clinician participation in research activities. Acad Med.

[19] Petrak J, Sember M, Granic D (2012). Assessing research productivity in Department of Internal Medicine, University of Zagreb, School of Medicine and University Hospital Centre Zagreb. Lijecnicki vjesnik.

[20] Hiscock H, Ledgerwood K, Danchin M, Ekinci E, Johnson E, Wilson A (2014). Clinical research potential in Victorian hospitals: the Victorian clinician researcher needs analysis survey. Intern Med J.

[21] Li X, Huang J, Zhang H (2008). An analysis of hospital preparedness capacity for public health emergency in four regions of China: Beijing, Shandong, Guangxi, and Hainan. BMC Public Health.

[22] The measures for the administration of the hospital grade. https://www.baidu.com/baidu?tn=monline_7_dg&ie=utf-8&wd=%E5%8C%BB%E9%99%A2%E5%88%86%E7%BA%A7%E7%AE%A1%E7%90%86%E6%A0%87%E5%87%86. Accessed 3 May 2020.

[23] Classification of Chinese Hospitals. https://en.wikipedia.org/wiki/Classification_of_Chinese_Hospitals. Accessed 3 May 2020.

[24] Hongwei F, Qi Z, Weidong H (2015). Analysis of influential factors of doctors’ research ability of affiliated hospitals of medical university. Chinese Hosp Manage.

[25] Magesa SM, Mwape B, Mboera LE (2011). Challenges and opportunities in building health research capacity in Tanzania: a case of the National Institute for Medical Research. Tanzania J Health Res.

[26] Howard AJ, Ferguson M, Wilkinson P, Campbell KL (2013). Involvement in research activities and factors influencing research capacity among dietitians. J Hum Nutr Dietetics.

[27] Barbón OG, Barriga SF, Cazorla AL, Cepeda LG (2018). Influence of seniority and total research hours on scientific production of university professors. Formación Universitaria.

[28] Geuna A, Nesta LJJ (2006). University patenting and its effects on academic research: the emerging European evidence. Res Policy.

[29] Owens B (2013). Long-term research: slow science. Nature.

[30] Krull JL, MacKinnon DP (2001). Multilevel modeling of individual and group level mediated effects. Multivariate Behav Res.

[31] Goldstein H. Multilevel Statistical Models. Chichester: Wiley; 2011.

[32] Hongwei F, Jingjie L, Zhixue D, Qi Z, Weidong H (2015). Establishment of research ability index system for assessing the affiliated hospitals of medical university. Chinese Hosp Manage.

[33] Hongwei F, Weidong H (2015). An Analysis of Influential Factors for Nurses’ Research Ability of Affiliated Hospitals of Medical University. Chinese Hosp Manag.

[34] Project 211 and 985. https://www.chinaeducenter.com/en/cedu/ceduproject211.php. Accessed 3 May 2020.

[35] Park E, Cho M, Ki CS (2009). Correct use of repeated measures analysis of variance. Korean J Lab Med.

[36] Clark PC Jr. The effects of multicollinearity in multilevel models. Dayton: Wright State University; 2013.

[37] Burlison JD, Quillivan RR, Kath LM, Zhou Y, Courtney SC, Cheng C, Hoffman JM. A multilevel analysis of US hospital patient safety culture relationships with perceptions of voluntary event reporting. J Patient Safety. 2020;16(3):187–93. 10.1097/PTS.0000000000000336 PMC5415419 27820722

[38] de la Fuente J, Caballero FF, Verdes E, Rodríguez-Artalejo F, Cabello M, de la Torre-Luque A, Sánchez-Niubó A, María Haro J, Ayuso-Mateos JL, Chatterji S: Are younger cohorts in the USA and England ageing better? Int J Epidemiol. 2019;48(6):1906–13. 10.1093/ije/dyz126 PMC6929538 31873752

[39] Yarris LM, Juve AM, Artino AR, Sullivan GM, Rougas S, Joyce B, Eva K (2014). Expertise, time, money, mentoring, and reward: systemic barriers that limit education researcher productivity-proceedings from the AAMC GEA Workshop. J Grad Med Educ.

[40] Khullar D, Chokshi DA, Kocher R, Reddy A, Basu K, Conway PH, Rajkumar R (2015). Behavioral economics and physician compensation--promise and challenges. New Engl J Med.

[41] Shaffer JG, Mather FJ, Wele M, Li J, Tangara CO, Kassogue Y, Srivastav SK, Thiero O, Diakite M, Sangare M (2019). Expanding research capacity in Sub-Saharan Africa through informatics, bioinformatics, and data science training programs in Mali. Front Genet.

[42] Byrne E, Donaldson L, Manda-Taylor L, Brugha R, Matthews A, MacDonald S, Mwapasa V, Petersen M, Walsh A (2016). The use of technology enhanced learning in health research capacity development: lessons from a cross country research partnership. Glob Health.

[43] Hojat M, Gonnella JS, Caelleigh AS (2003). Impartial judgment by the “gatekeepers” of science: fallibility and accountability in the peer review process. Adv Health Sci Educ.

[44] Oyama Y, Fukahori H, Miyashita M, Narama M, Kono A, Atogami F, Kashiwagi M, Okaya K, Takamizawa E, Yoshizawa T (2015). Cross-sectional online survey of research productivity in young Japanese nursing faculty. Japan J Nurs Sci.

[45] Smirnoff M, Ramirez M, Kooplimae L, Gibney M, McEvoy MD (2007). Nurses’ attitudes toward nursing research at a metropolitan medical center. Appl Nurs Res.

[46] Henly SJ, Struthers R, Dahlen BK, Ide B, Patchell B, Holtzclaw BJ (2006). Research careers for American Indian/Alaska native nurses: pathway to elimination of health disparities. Am J Public Health.

[47] Malmgren RD, Ottino JM, Nunes Amaral LA (2010). The role of mentorship in protege performance. Nature.

[48] Way SF, Morgan AC, Larremore DB, Clauset A (2019). Productivity, prominence, and the effects of academic environment. Proc Natl Acad Sci USA.

[49] McGee R, Saran S, Krulwich TA (2012). Diversity in the biomedical research workforce: developing talent. Mount Sinai J Med.

[50] Feng S, Han M, Lai L, Wang SC, Liu JP (2017). Research capacity at traditional Chinese medicine (TCM) centers in China: a survey of clinical investigators. Evid Based Complement Altern Med.

[51] Varshney D, Atkins S, Das A, Diwan V (2016). Understanding collaboration in a multi-national research capacity-building partnership: a qualitative study. Health Res Policy Syst.

[52] Gonzalez Block MA, Mills A (2003). Assessing capacity for health policy and systems research in low and middle income countries. Health Res Policy Syst.

[53] Schlenker MB, Manalo E, Wong AM (2013). Research productivity of Canadian ophthalmology departments in top 10 ophthalmology and vision science journals from 2001 to 2010. Can J Ophthalmol.

[54] Eddleston M (1999). Encouraging high-quality clinical research in the tropics. Lancet.

[55] Marshall JC (2017). Global collaboration in acute care clinical research: opportunities, challenges, and needs. Crit Care Med.

[56] Mahmood S, Hort K, Ahmed S, Salam M, Cravioto A (2011). Strategies for capacity building for health research in Bangladesh: Role of core funding and a common monitoring and evaluation framework. Health Res Policy Syst.

[57] Liu Y, Yang Z, Fan D (2016). Professional title promotion among clinicians: a cross-sectional survey. Lancet.

[58] Zhang Z (2015). Statistical analysis on health management of National Social Science Fund Projects. Sci Technol Manage Res.

[59] Eschelman DJ, Sullivan KL, Parker L, Levin DC (2000). The relationship of clinical and academic productivity in a university hospital radiology department. Am J Roentgenol.

[60] Huang Y, Pang S-K, Yu S (2018). Academic identities and university faculty responses to new managerialist reforms: experiences from China. Studies Higher Educ.

[61] Zhou F, Hao Y, Guo H, Liu H (2016). Attitude, knowledge, and practice on evidence-based nursing among registered nurses in traditional Chinese medicine hospitals: a multiple center cross-sectional survey in China. Evid Based Complement Altern Med.

[62] Lin CF, Huang CI, Yang CM, Lu MS (2019). The relationship between work environment satisfaction and retention intention among nursing administrators in Taiwan. J Nurs Res.

[63] Fei W, Jingbo W, Zhanpeng Y (2019). Evaluation on hospital scientific research performance based on application in data envelopment analysis method. Hosp Admin J Chin PLArmy.

