# Supplementary material for: Health research capacity of professional and technical personnel in a first-class tertiary hospital in northwest China: multilevel repeated measurement, 2013–2017, a pilot study
Source: Health Res Policy Syst. 2020 Sep 17;18:103. doi: 10.1186/s12961-020-00616-7 (PMC7499869; doi:10.1186/s12961-020-00616-7)
Supplement: Supplementary file 1 — Additional file 1: eTable 1 Scientific research capability evaluation system. [file 12961_2020_616_MOESM1_ESM.docx]

eTable 1 Scientific Research Capability Evaluation System

| First-level indicators | Second-level indicators | Weight  (points) |
| --- | --- | --- |
| Research projects | 1. Holding 863 Program, 973 Program, National Science and Technology Support Program, The National Natural Science Foundation of China Key Projects, The National Science Fund for Distinguished Young Scholars | 9.18 |
|  | 2. Holding General Program, Young Scientists Fund or Cooperation Program of the National Natural Science Foundation of China | 7.14 |
|  | 3. Holding 863 Program, 973 Program, sub-project of National Science and Technology Support Program and etc. (more than 200,000 CNY) | 5.10 |
|  | 4. Holding ministerial-level projects and provincial key projects | 4.08 |
|  | 5. Holding 863 Program, 973 Program, sub-project of National Science and Technology Support Program and etc. (less than 200,000 CNY) | 3.06 |
|  | 6. Holding Department-level key projects | 2.04 |
|  | 7. Holding Department-level general projects and Projects of Civil Society Organizations | 1.02 |
|  | 8. Holding other projects | 0.61 |
|  | 9. Participating in General Program, Young Scientists Fund, Cooperation Program of the National Natural Science Foundation of China | 0.41 |
| Research awards | 10. From the Ministry of Education and National Health Commission of the People's Republic of China, Chinese medical science and Technology Award | 9.18 |
|  | 11. The first prize of the Provincial Government | 7.14 |
|  | 12. The second prize of Provincial Government | 6.12 |
|  | 13. The third prize of the Provincial Government | 4.08 |
|  | 14. The first prize of Department- and Bureau-level Award | 3.06 |
|  | 15. The second prize of Department- and Bureau-level Award | 2.04 |
|  | 16. The third prize of Department- and Bureau-level Award | 1.02 |
|  | 17. Award of Civil Society Organizations | 0.61 |
| Patents | 18. Won one national invention patent | 6.12 |
| Monographs | 19. The editor in chief | 2.04 |
|  | 20. The deputy editor | 0.61 |
|  | 21. Wrote some books | 0.20 |
| Published scientific papers | 22. One Scientific Citation Index Paper with IF <8 | 9.18 |
|  | 23. One Scientific Citation Index Paper with 3＜IF ≤8 | 6.12 |
|  | 24. One Scientific Citation Index Paper with 1＜IF ≤3 | 4.08 |
|  | 25. One Scientific Citation Index Paper with IF ≤1 | 1.02 |
|  | 26. One Chinese paper | 0.61 |
| Trained students | 27. One student for Ph.D | 3.06 |
|  | 28. One postgraduate | 1.02 |

CNY, China Yuan; IF, impact factor; Ph.D, Doctor of Philosophy
