# Supplementary material for: Health research capacity of professional and technical personnel in a first-class tertiary hospital in northwest China: multilevel repeated measurement, 2013–2017, a pilot study
Source: Health Res Policy Syst. 2020 Sep 17;18:103. doi: 10.1186/s12961-020-00616-7 (PMC7499869; doi:10.1186/s12961-020-00616-7)
Supplement: Supplementary file 2 — Additional file 2: eTable 2 Multilevel variance inflation factors of variables in the multilevel repeated measurement model. [file 12961_2020_616_MOESM2_ESM.docx]

eTable 2 Multilevel variance inflation factors of variables in the multilevel repeated measurement model

|  | Sex | Age | Ethnic | Degree | Graduate school | Technical type | Title | Department | With administrative position | Research fund |
| --- | --- | --- | --- | --- | --- | --- | --- | --- | --- | --- |
| Sex | 1.105 | 0.256 | -0.004 | -0.166 | 0.044 | -0.236 | -0.034 | 0.040 | -0.007 | -0.032 |
| Age | 0.256 | 4.202 | -0.077 | -0.824 | -0.105 | 0.043 | -3.351 | -0.108 | -0.193 | 0.072 |
| Ethnic | -0.004 | -0.077 | 1.018 | 0.086 | -0.093 | -0.052 | 0.162 | -0.001 | 0.009 | -0.060 |
| Degree | -0.166 | -0.824 | 0.086 | 2.730 | -1.655 | -0.424 | 0.268 | -0.093 | -0.038 | 0.532 |
| Graduate school | 0.044 | -0.105 | -0.093 | -1.655 | 2.274 | -0.118 | 0.257 | 0.179 | -0.181 | -0.099 |
| Technical type | -0.236 | 0.043 | -0.052 | -0.424 | -0.118 | 1.429 | 0.172 | -0.463 | -0.030 | -0.187 |
| Title | -0.034 | -3.351 | 0.162 | 0.268 | 0.257 | 0.172 | 4.357 | -0.074 | -0.567 | -0.509 |
| Department | 0.040 | -0.108 | -0.001 | -0.093 | 0.179 | -0.463 | -0.074 | 1.208 | 0.227 | 0.019 |
| With administrative position | -0.007 | -0.193 | 0.009 | -0.038 | -0.181 | -0.030 | -0.567 | 0.227 | 1.760 | -0.571 |
| Research fund | -0.032 | 0.072 | -0.060 | 0.532 | -0.099 | -0.187 | -0.509 | 0.019 | -0.571 | 1.580 |

Multilevel variance inflation factors are on the diagonals and highlighted
